# Prognostic factors and predictive models for patients with lung large cell neuroendocrine carcinoma: Based on SEER database

**DOI:** 10.1111/crj.13752

**Published:** 2024-04-12

**Authors:** Wenqiang Li, Qian Huang, Xiaoyu He, Qian He, Qun Lai, Quan Yuan, Zhiping Deng

**Affiliations:** ^1^ Zigong First People's Hospital Zigong City Sichuan Province China; ^2^ Dazhou Dachuan District People's Hospital Dazhou Sichuan Province China; ^3^ Sichuan North Medical College Nanchong Sichuan Province China; ^4^ West China Second Hospital of Sichuan University Sichuan Province China; ^5^ The first hospital of Jilin University Jilin Province People's Republic of China

**Keywords:** lung large cell neuroendocrine carcinoma, nomogram, population distribution, predictors, prognosis

## Abstract

**Background:**

Lung Large cell neuroendocrine carcinoma (LCNEC) is a rare, aggressive, high‐grade neuroendocrine carcinoma with a poor prognosis, mainly seen in elderly men. To date, we have found no studies on predictive models for LCNEC.

**Methods:**

We extracted data from the Surveillance, Epidemiology, and End Results (SEER) database of confirmed LCNEC from 2010 to 2018. Univariate and multivariate Cox proportional risk regression analyses were used to identify independent risk factors, and then we constructed a novel nomogram and assessed the predictive effectiveness by receiver operating characteristic (ROC) curves, calibration curves, and decision curve analysis (DCA).

**Results:**

A total of 2546 patients with LCNEC were included, excluding those diagnosed with autopsy or death certificate, tumor, lymph node, metastasis (TNM) stage, tumor grade deficiency, etc., and finally, a total of 743 cases were included in the study. After univariate and multivariate analyses, we concluded that the independent risk factors were N stage, intrapulmonary metastasis, bone metastasis, brain metastasis, and surgical intervention. The results of ROC curves, calibration curves, and DCA in the training and validation groups confirmed that the nomogram could accurately predict the prognosis.

**Conclusions:**

The nomogram obtained from our study is expected to be a useful tool for personalized prognostic prediction of LCNEC patients, which may help in clinical decision‐making.

## Graphical abstract

A practical visualization model for prognosis of lung large cell neuroendocrine carcinoma: based on the SEER database.

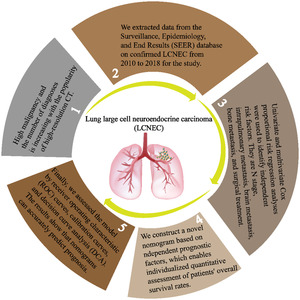

## 1 INTRODUCTION

Lung large cell neuroendocrine carcinoma (LCNEC) is a rare, aggressive tumor with a poor prognosis. It is generally categorized in the non‐small cell lung cancer (NSCLC) group because it is currently considered a neuroendocrine subtype of large cell lung cancer.^1^ However, it is also a member of the group of lung neuroendocrine tumors, which are predominantly seen in older men and account for approximately 15% of all lung neuroendocrine tumors and 3% of all lung cancers.^2^, ^3^ The poor survival outcome of LCNEC is mainly due to local recurrence and distant metastases, and its five‐year survival rates range from 15% to 57%.^4^ A previous study showed that the total age‐adjusted incidence of LCNEC during 2000–2013 was 0.3/100000, of which 0.4/100000 and 0.3/100000 were in men and women,^5^ respectively, and the incidence was on the rise during this period, with 5‐year lung cancer‐specific survival (LCSS) and overall survival (OS) of 20.7% and 16.7%.

LCNEC has a poor prognosis, low incidence, and is mostly disseminated. Due to its epidemiology and unique biology, there is an urgent need for multicenter, large‐sample prognostic studies. The SEER database is an open tumor database in the U.S., originating from multiple medical institutions and covering about 30% of the U.S. population. It can provide large sample data for tumor‐related studies. Nomograms are now widely used to assess the prognosis of cancer patients because of their quantitative and intuitive nature.^6^, ^7^ Therefore, we constructed a novel nomogram using the SEER database, which can be personalized to predict prognosis and help guide clinical decisions.

## 2 MATERIALS AND METHODS

### 2.1 Patients data collection

**FIGURE 1:**
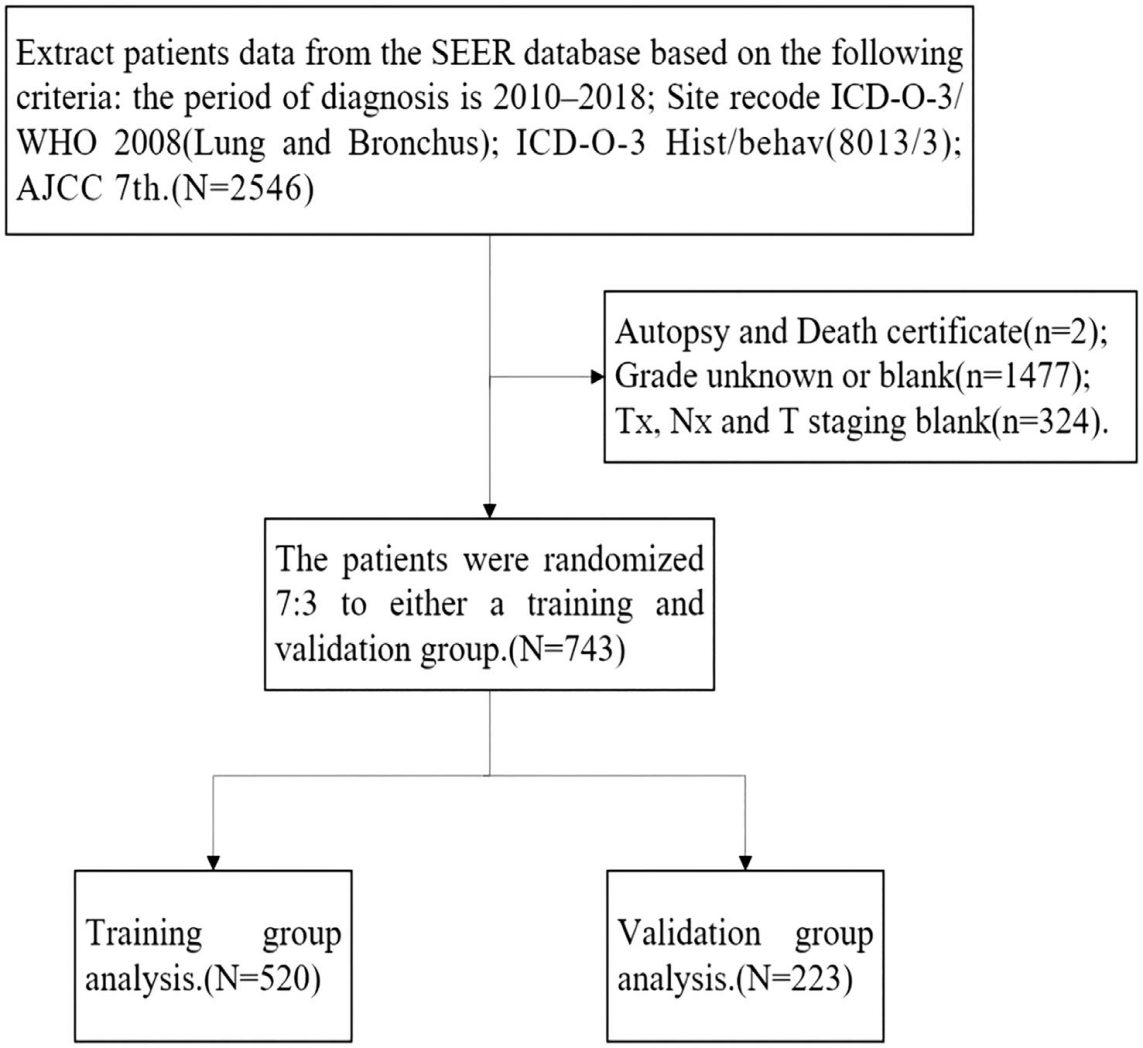
The patients screening process.

The SEER database is an open database containing frequency and survival data. We selected the incidence‐SEER Research Plus Data 18 Registries, Nov 2020 Sub [2000–2018] database and extracted patients diagnosed with LCNEC in the database from 2010–2018 as study data. (1) Inclusion criteria: the period of diagnosis is 2010–2018; Site recode ICD‐O‐3/WHO 2008 (Lung and Bronchus); ICD‐O‐3 Hist/behav (8013/3); AJCC 7th; (2) exclusion criteria: tumor grade blank or unknown; TX, NX, T stage blank; patients diagnosed with autopsy or death certificate (Figure 1). The following variables were collected: (1) Demographic variables, including age, gender, race, and marital status. (2) Clinical information, including tumor pathological grade, TNM stage, distant metastases (lung, liver, bone, brain), radiotherapy, chemotherapy, and surgical treatment.

In determining the sample size for multivariate cox regression, we are usually based on the 10EPV (Events Per Variable, each variable eventually included in the regression model has 10 positive events, and if there are more positive events in the study, it should also be considered that the data of negative events meet 10EPV) method, which is widely used in model building studies.^8^ We estimate that eventually, about 9–11 variables enter the final multivariate cox regression. According to the 10EPV principle, positive events were greater than the number of negative event cases, so the number of negative events in the training group was at least 90 or 110. If randomly divided into the training group and the validation group at a ratio of 7:3, the number of negative events in the training group was about 126 cases (greater than 110 cases), so the sample size of the model we constructed was sufficient. Finally, 743 patients were included in this study and were randomized in a 7:3 ratio into a training group (70%) and a validation group (30%). We constructed and internally validated the nomogram with the training group, followed by external validation by the validation group.

### 2.2 Statistical analysis

In this study, all statistical analyses were performed with R software (version 4.2.1), and a *P* value < 0.05 (bilateral) was considered statistically significant. All patients were randomly divided into training and validation groups, and the distribution of variables between the two groups was compared using the chi‐square test.

For prognostic factor analysis, we used univariate COX regression analysis to identify factors associated with OS in patients with LCNEC. Then, significant variables (*P* < 0.05) were included, and a “forward LR” multivariate Cox regression analysis was used to further identify independent prognostic factors. We created a prognostic nomogram to predict 12, 24, and 36 months OS based on independent factors and calculated individual risk scores. We evaluated the efficacy of the model in three dimensions. First, we plotted the ROC curves over time and calculated the corresponding area under the curve (AUC) over time to assess discrimination. Second, calibration curves were plotted to assess the predictive accuracy of the nomogram. Finally, the DCA was performed to assess the model's efficacy in clinical practice.

## 3 RESULTS

### 3.1 Characteristics of patients

A total of 743 patients were included. The age was predominantly 50–80 years, the majority were male 404 (54.4%), white 624 (84.0%), and grade III‐IV 722 (97.2%) patients. All included patients were randomized to the training group (n = 520) and the validation group (n = 223) at a ratio of 7:3, while the chi‐square test demonstrated no significant differences in all factors between the two subgroups (Table 1).

**TABLE 1:** Baseline clinical characteristics of LCNEC patients.

	Training	Validation	Overall	χ^2^	P
	(N = 520)	(N = 223)	(N = 743)		
Sex				1.007	0.315
Female	244 (46.9%)	95 (42.6%)	339 (45.6%)		
Male	276 (53.1%)	128 (57.4%)	404 (54.4%)		
Age				0.07	0.791
<65	240 (46.2%)	106 (47.5%)	346 (46.6%)		
>65	280 (53.8%)	117 (52.5%)	397 (53.4%)		
Marital				0.735	0.391
Married	244 (46.9%)	113 (50.7%)	357 (48.0%)		
Other	276 (53.1%)	110 (49.3%)	386 (52.0%)		
Race				0.671	0.715
Black	64 (12.3%)	30 (13.5%)	94 (12.7%)		
White	440 (84.6%)	184 (82.5%)	624 (84.0%)		
Other	16 (3.1%)	9 (4.0%)	25 (3.4%)		
Grade				2.385	0.122
I‐II	11 (2.1%)	10 (4.5%)	21 (2.8%)		
III‐IV	509 (97.9%)	213 (95.5%)	722 (97.2%)		
T				0.186	0.666
T0‐T2	309 (59.4%)	137 (61.4%)	446 (60.0%)		
T3‐T4	211 (40.6%)	86 (38.6%)	297 (40.0%)		
N				5.781	0.123
N0	262 (50.4%)	128 (57.4%)	390 (52.5%)		
N1	68 (13.1%)	23 (10.3%)	91 (12.2%)		
N2	137 (26.3%)	59 (26.5%)	196 (26.4%)		
N3	53 (10.2%)	13 (5.8%)	66 (8.9%)		
Metastasis					
Bone				0.016	0.899
No/Unknown	465 (89.4%)	198 (88.8%)	663 (89.2%)		
Yes	55 (10.6%)	25 (11.2%)	80 (10.8%)		
Liver				0.047	0.827
No/Unknown	473 (91.0%)	201 (90.1%)	674 (90.7%)		
Yes	47 (9.0%)	22 (9.9%)	69 (9.3%)		
Brain				1.078	0.299
No/Unknown	453 (87.1%)	201 (90.1%)	654 (88.0%)		
Yes	67 (12.9%)	22 (9.9%)	89 (12.0%)		
Lung				0.01	0.919
No/Unknown	487 (93.7%)	210 (94.2%)	697 (93.8%)		
Yes	33 (6.3%)	13 (5.8%)	46 (6.2%)		
Radiation				1.674	0.196
No/Unknown	327 (62.9%)	152 (68.2%)	479 (64.5%)		
Yes	193 (37.1%)	71 (31.8%)	264 (35.5%)		
Chemotherapy				1.508	0.219
No/Unknown	234 (45.0%)	112 (50.2%)	346 (46.6%)		
Yes	286 (55.0%)	111 (49.8%)	397 (53.4%)		
Surgery				0.014	0.905
No	249 (47.9%)	105 (47.1%)	354 (47.6%)		
Yes	271 (52.1%)	118 (52.9%)	389 (52.4%)		

**Grade**: I, well differentiated; II, moderately differentiated; III, poorly differentiated; IV, undifferentiated. ***P***: values calculated by chi‐square test.

### 3.2 Prognostic factors for LCNEC patients

Univariate and multivariate Cox regression analyses were used to screen for robust prognostic factors in the grouped training group, and the results showed that N stage, intrapulmonary metastasis (yes or no), brain metastasis (yes or no), bone metastasis (yes or no), and surgery at the primary site (yes or no) were independent prognostic factors (Table 2).

**TABLE 2:** Univariate and multivariate cox analyses in LCNEC patients.

	Univariate analysis		Multivariate analysis	
	HR(95%CI)	P^$^	HR(95%CI)	P^^^
Sex				
Female	Reference		Reference	
Male	1.301(1.066–1.588)	0.009	1.141(0.930–1.401)	0.204
Age				
<65	Reference			
>65	1.213(0.993–1.480)	0.058		
Marital				
Married	Reference			
Other	0.976(0.801–1.190)	0.815		
Race				
Black	Reference			
White	1.132(0.833–1.536)	0.428		
Other	1.320(0.714–2.441)	0.375		
Grade				
I‐II	Reference			
III‐IV	1.530(0.724–3.232)	0.265		
T				
T0‐T2	Reference		Reference	
T3‐T4	2.224(1.820–2.717)	<0.001	1.219(0.966–1.538)	0.094
N				
N0	Reference		Reference	
N1	2.100(1.552–2.842)	<0.001	1.540(1.118–2.123)	<0.001
N2	3.004(2.366–3.816)	<0.001	1.711(1.296–2.257)	<0.001
N3	4.065(2.949–5.605)	<0.001	2.101(1.464–3.014)	<0.001
Metastasis				
Bone				
No/Unknown	Reference		Reference	
Yes	3.403(2.527–4.582)	<0.001	1.633(1.184–2.252)	0.002
Liver				
No/Unknown	Reference		Reference	
Yes	2.965(2.168–4.054)	<0.001	1.031(0.723–1.470)	0.864
Brain				
No/Unknown	Reference		Reference	
Yes	3.219(2.450–4.229)	<0.001	2.537(1.857–3.466)	<0.001
Lung				
No/Unknown	Reference		Reference	
Yes	3.803(2.636–5.486)	<0.001	1.948(1.325–2.864)	<0.001
Radiation				
No/Unknown	Reference		Reference	
Yes	1.784(1.457–2.185)	<0.001	0.818(0.641–1.044)	0.107
Chemotherapy				
No/Unknown	Reference			
Yes	1.103(0.902–1.348)	0.336		
Surgery				
No	Reference		Reference	
Yes	0.263(0.214–0.325)	<0.001	0.437(0.328–0.582)	<0.001

**Grade**: I, well differentiated; II, moderately differentiated; III, poorly differentiated; IV, undifferentiated. ***P*** ^***$***^: values calculated by univariate COX regression analysis. ***P*** ^***^***^: values calculated by multivariate COX regression analysis.

### 3.3 Generation and validation of the prognostic nomograms

**FIGURE 2:**
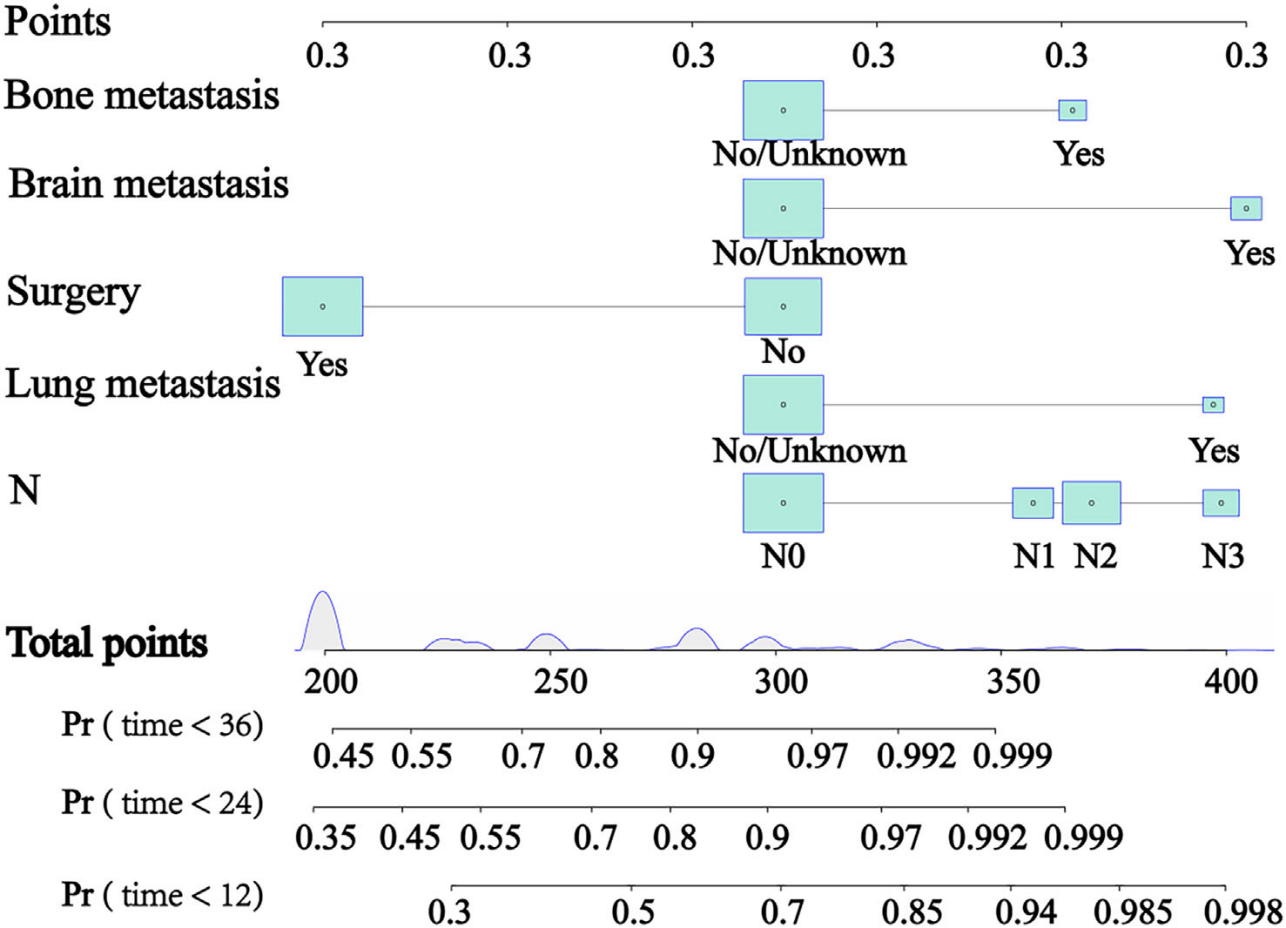
A prognostic nomogram for predicting the OS of LCNEC for the 12, 24, and 36 months.

**FIGURE 3:**
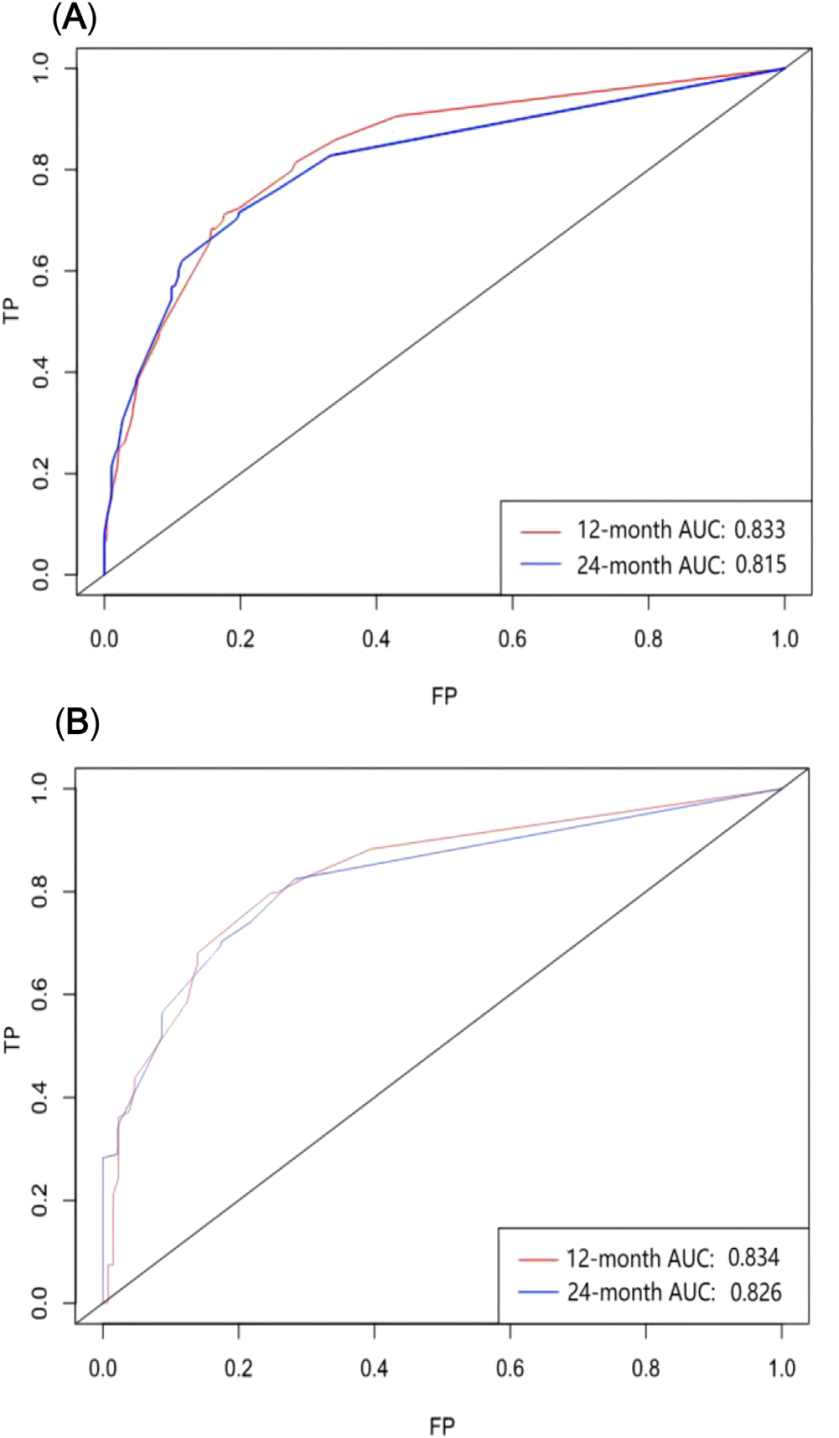
The receiver operating characteristic curve of the nomogram for the 12 and 24 months in the training set (A) and validation set (B).

**FIGURE 4:**
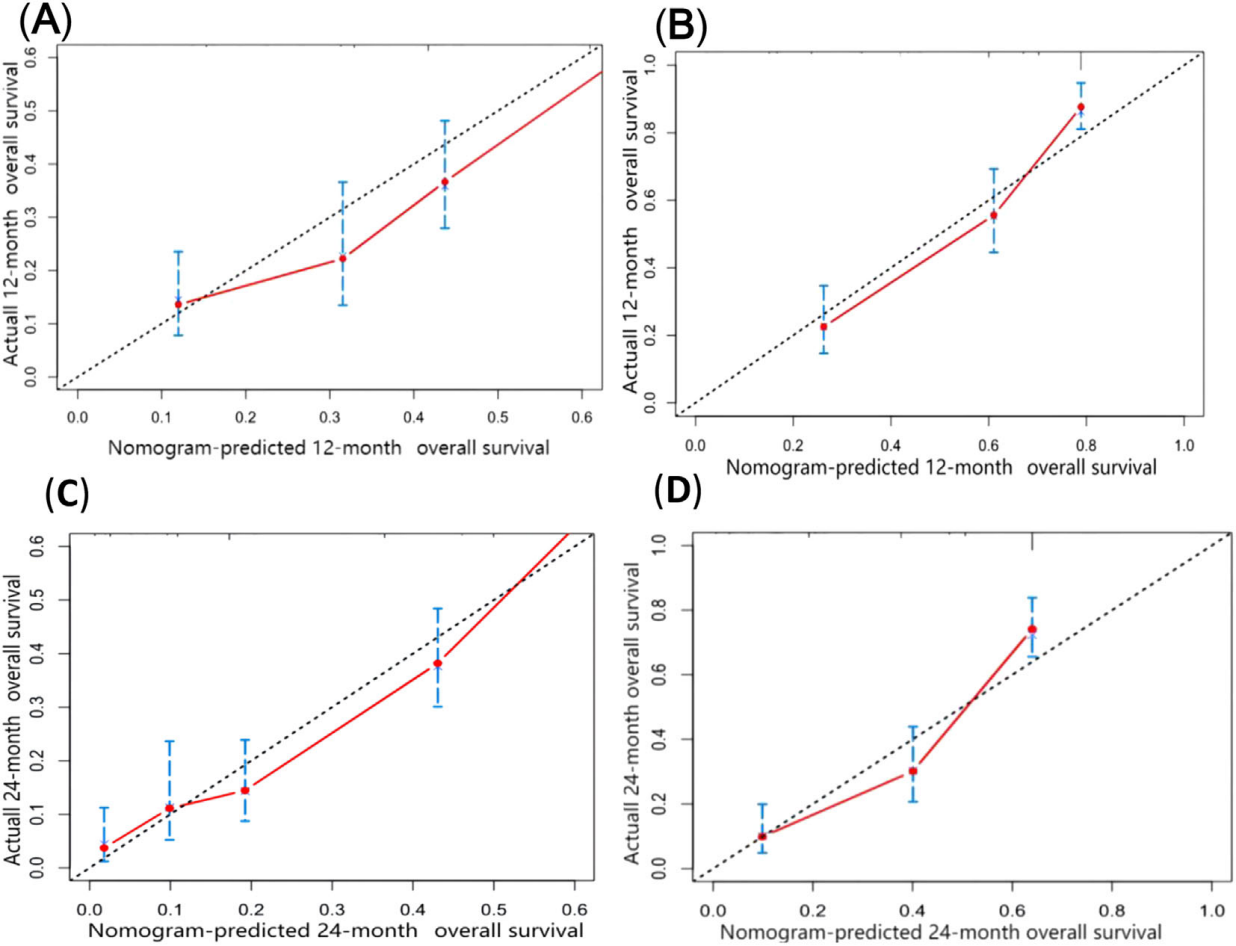
The calibration curves of the nomogram for the 12 and 24 months in the training set (A, C) and validation set (B, D).

**FIGURE 5:**
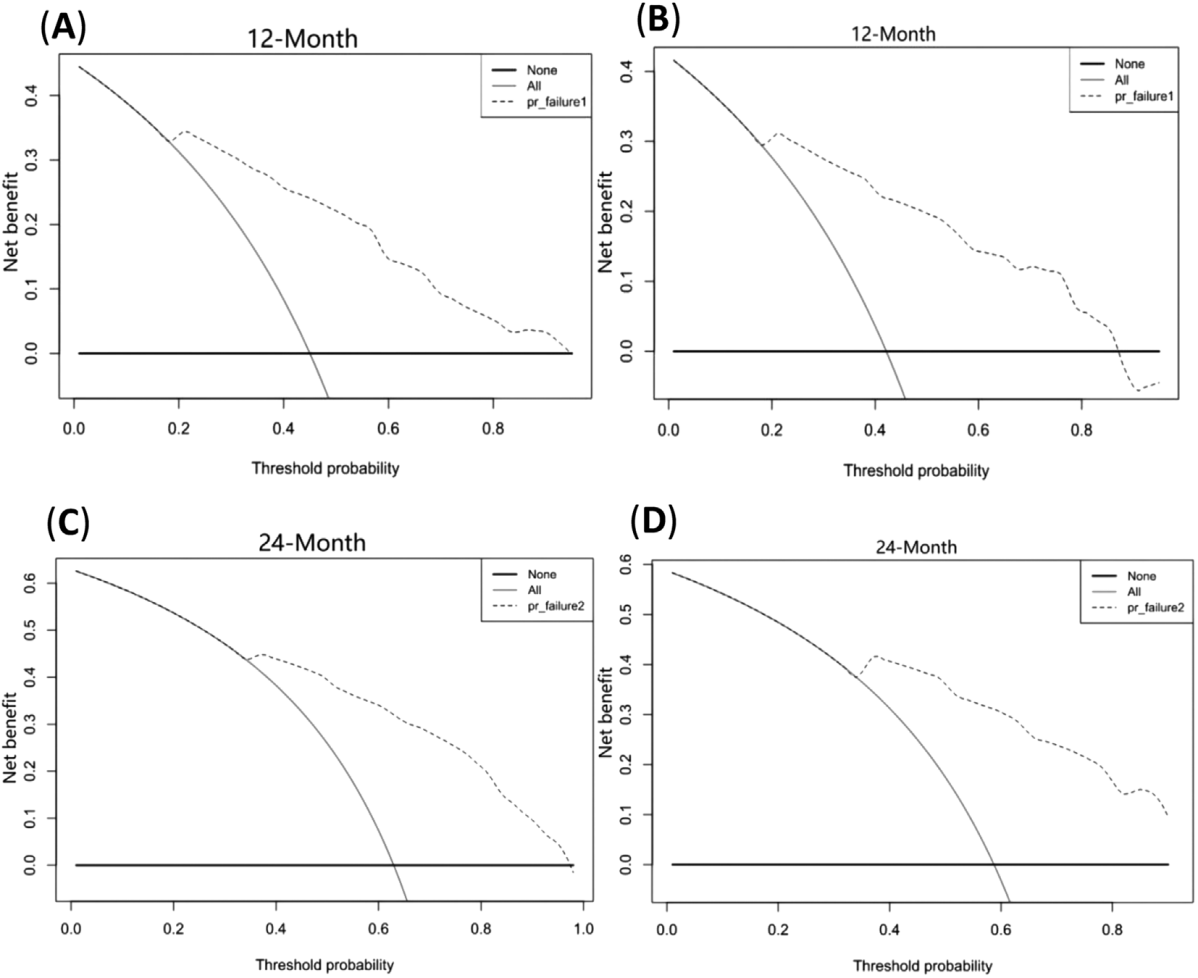
The decision curve analysis of the nomogram for the 12 and 24 months in the training set (A, C) and validation set (B, D).

Based on the above five prognostic factors, a novel nomogram predicting OS at 12, 24, and 36 months in LCNEC patients was developed (Figure 2). Then, the ROC diagram showed that the AUC of 12 and 24 months nomogram reached 0.833 and 0.815 in the training group (Figure 3A) and 0.834 and 0.826 in the validation group (Figure 3B), respectively, with a good discriminatory ability for predicting, and the validation group was better than the training group. The calibration curves were plotted to assess the 12 and 24 months OS prediction nomogram efficacy, and the results showed strong agreement between the nomogram‐predicted OS and the actual outcomes in the training group (Figure 4A,C) and the validation group (Figure 4B, D). In addition, The DCA also determined that the nomogram had good performance in clinical practice (Figure 5A‐D).

## 4 DISCUSSION

We took advantage of the scientific, reliable, and abundant samples of the SEER database to complete this study. Most of the previous studies have discussed the prognostic factors associated with LCNEC,^5^, ^9^, ^10^, ^11^ and there is a lack of quantitative assessment tools. In this study, a quantitative prognostic tool for LCNEC was constructed for the first time, and it was verified to have good differential ability, predictive accuracy, and clinical efficacy.

Currently, the World Health Organization (WHO) classification defines LCNEC as NSCLC with histopathological features and immunohistochemical expression of neuroendocrine carcinoma. Some studies have found that it is composed of different subgroups with genomic features of small cell lung cancer (SCLC), NSCLC (mainly adenocarcinoma), and rare highly proliferative carcinoid tumors.^7^ It has a strong ability to distant metastasis, resulting in high mortality. Therefore, It is necessary for us to clarify its prognostic factors, which helpfully make accurate clinical decisions. In this study, we generated a novel nomogram to personalize the prediction of OS in LCNEC patients based on data obtained from the SEER database. We found that N stage, bone metastasis (yes or no), brain metastasis (yes or no), intrapulmonary metastasis (yes or no), and surgery at the primary site (yes or no) were independent prognostic factors. Among them, Surgery at the primary site favors the prognosis and prolongs their OS. Based on five key variables, the nomogram could calculate scores to quantitatively assess the prognosis. In addition, we plotted the time‐dependent ROC curves. According to the corresponding time‐dependent AUC values, we know that the model has good discriminatory ability. We also plotted calibration curves and DCA to confirm that the nomogram had good consistency and clinical practicability. Based on the above results, our model can provide good guidance for further clinical evaluation and intervention.

Nowadays, for pulmonary neuroendocrine tumors, the International Association for the Study of Lung Cancer recommends the application of TNM staging to predict their prognosis.^9^ Among them, LCNEC exhibits a high rate of lymph nodes (60%–80%) and distant metastases (40%), similar to SCLC.^12^ The 5‐year OS of LCNEC varies by clinical staging (CS), with stages I‐IV ranging from 33%–62%, 18%–75%, 8%–45%, and 0–2.6%, respectively.^5^, ^13^, ^14^, ^15^, ^16^, ^17^ Meantime, the median overall survival (mOS) also varies, with stages I‐IV ranging from 44–105, 19–28, 14–23, and 6–10.2 months, respectively.^5^, ^18^, ^19^ However, the effects of T, N, and M stages on different subtypes of lung cancer are also different. T and N‐staging are independent factors of OS in SCLC patients. The gap in OS between T_2_ and T_3–4_ staging is greater than that between T_1_ and T_2_ staging, and the difference in the effect of N_0_ versus N_1_ staging on OS is larger, whereas the difference in the effect of N_1_ versus N_2_ staging is smaller.^20^ Squamous carcinoma, a common NSCLC, also has T‐staging and N‐staging as independent influences. There is a large difference in OS between N_0_ and N_1_ stages, and among the T_1_‐T_4_ stages, T_3_, and T_4_ stages have the largest difference in OS, while T_2_ and T_3_ have the smallest difference.^21^ In comparison to other types of lung cancer, T, N, and M staging each had a different impact on OS in patients with LCNEC. In this study, we found that the T stage had no significant influence on OS in the TNM stage of LCNEC patients, while the N and M stages (bone, lung, and brain metastases) were independent influences on OS. Among the N_0_‐N_3_ staging, the gap in OS between N_0_ and N_1_ staging was the largest, and N_1_ and N_2_ were the smallest. Of all the factors of significance, M staging had the greatest impact. Of these, brain metastases (yes or no) had the greatest impact, suggesting that the occurrence of brain metastases has the worst prognosis, which may be related to the higher rate of brain metastasis in patients with stage IV LCNEC.^19^ Moreover, we also found that the result when performing univariate analysis showed that liver metastases were unfavorable for prognosis, but multivariate analysis showed no clear effect on the prognosis. This is different from the results of another study, so we need to continue exploring.^22^ Similar to the results of another study,^23^ the result of this study also showed no clear correlation between tumor grade and patient OS.

For the treatment of patients with LCNEC, the National Cancer Control Network (NCCN) recommends treatment according to NSCLC guidelines. However, because both LCNEC and SCLC are high‐grade neuroendocrine tumors, they are usually treated with the same chemotherapy regimen.^24^, ^25^, ^26^ Currently, response rates of 50%–80% have been demonstrated with platinum/etoposide regimens in patients with extensive stage SCLC,^27^, ^28^, ^29^ but the response rate to the same regimen is much lower in LCNEC.^19^, ^24^, ^25^, ^26^ One study showed that chemotherapy was beneficial and radiotherapy had no significant benefit for the prognosis,^10^ while another study showed that radiotherapy and chemotherapy were beneficial for OS in LCNEC patients.^11^ A retrospective study showed that surgery combined with chemotherapy was the best treatment for patients with stage I, II, and III LCNEC, and for stage IV patients, chemotherapy alone was more effective than other treatments.^30^ Lowczak et al demonstrated that patients with radical, negative surgical margins, lower CS, negative lymph nodes, and tumor size ≤4 cm have significantly better survival prognosis.^18^ These results suggest that surgical interventions are positively associated with patient prognosis. However, the benefits of chemotherapy and radiotherapy for LCNEC are controversial. Nevertheless, the sample sizes in these studies were small. In our large sample study, there was no clear benefit of radiotherapy or chemotherapy on the prognosis. However, there is a significant benefit of surgery on OS, which is similar to the previous results.^18^

In addition to radiotherapy and chemotherapy, emerging treatments for LCNEC are immunologic and targeted therapies.^31^, ^32^ Moreover, cyclic RNA is an emerging biomarker that regulates cancer proliferation, apoptosis, migration, and invasion through multiple mechanisms. Its aberrant expression is present in almost all types of cancers.^33^ It has guiding significance for cancer diagnosis and prognosis and is expected to serve as a target for cancer treatment.^34^ Unfortunately, the SEER database does not have relevant data at this time and is to be updated at a later date. Of course, there are still some shortcomings in this study. Some factors that influence prognosis, such as underlying disease, Ki67, PD‐L1 expression, and details of chemotherapy and radiotherapy, are not recorded in the SEER database, or a large amount of data is missing. Second, internal bias and limited significance are inevitable, constrained by retrospective data analysis and non‐randomization. Therefore, in the future, we will try to include more covariates and extend the follow‐up time in the clinic to further improve the predictive value of the model.

## 5 CONCLUSION

In this study, we included basic information and treatment regimens in addition to TNM staging to analyze and construct a predictive model that more accurately calculates OS in patients with LCNEC. In addition, we verified that it has good discriminatory ability, predictive accuracy, and clinical practicability.

## AUTHOR CONTRIBUTIONS

Wenqiang Li and Qian Huang designed the study. Qian He and Xiaoyu He extracted data from the SEER database. Wenqiang Li and Quan Yuan analyzed the data. Qun Lai and Xiaoyu He drew figures and tables. Qian Huang wrote the manuscript. Zhiping Deng reviewed the manuscript.

## CONFLICT OF INTEREST STATEMENT

The authors declare that the research was conducted in the absence of any commercial or financial relationships that could be construed as a potential conflict of interest.

## ETHICS STATEMENT

We signed the SEER Study Data Agreement to access SEER information using reference number 20225‐Nov2021. We performed study methods following approved guidelines to access data provided by the SEER database. The Office of Human Research Protections considers data analysis to be non‐human subjects studied by the U.S. Department of Health and Human Services because they are publicly available and de‐identified. Therefore, it does not require any approval from the Institutional Review Board.

## Data Availability

The data that support the findings of this study are available in Surveillance, Epidemiology, and End Results at https://seer.cancer.gov/data-software/, reference number 20225‐Nov2021. These data were derived from the following resources available in the public domain: National Cancer Institute, https://seer.cancer.gov/data-software/.
